# Down‐regulated IL36RN expression based on peripheral blood mononuclear cells and plasma of periodontitis patients and its clinical significance

**DOI:** 10.1002/jcla.23899

**Published:** 2021-07-17

**Authors:** Yue Zhou, Yufu Liang

**Affiliations:** ^1^ Department of Stomatology Affiliated Hospital of Beihua University Jilin China

**Keywords:** cytokines, IL36RN, PBMC, periodontitis, plasma

## Abstract

**Background:**

The role of IL‐36 receptor antagonist (IL36RN), a mutated gene expression of IL‐36 in periodontitis patients with peripheral blood mononuclear cells (PBMC) and plasma remains to be undetermined.

**Materials and methods:**

Our study discovered the IL36RN expression through GEO public databases and further validated by PBMC and plasma of periodontitis patients and healthy participants. A total of 194 participants of public datasets, consisting of 97 cases of periodontitis and 97 cases of healthy control were retrospectively evaluated and explored the gene enrichment pathways and clinical significance of IL36RN expression accompanied by three different cytokines. Furthermore, the clinical significance of IL36RN was evaluated in mild‐to‐severe patients of periodontitis by the receiver operating curve (ROC) using the area under the curve (AUC).

**Results:**

IL36RN expressions were notably down‐regulated in PBMC and plasma of periodontitis patients. Further, a positive correlation of IL36RN expression was significantly observed between PBMC and plasma of periodontitis patients while IL36RN expression was negatively correlated to serum‐based three different cytokines of periodontitis patients. Meanwhile, the ROC‐AUCs achieved a significantly higher range from 0.80 to 0.87 with PBMC of mild‐to‐severe and moderate‐to‐severe periodontitis patients whereas similar patients with plasma obtained a significant AUC range from 0.73 to 0.83.

**Conclusion:**

IL36RN can distinctively be detectable in periodontitis patients with PBMC and plasma, which can act as a down‐regulated mutated gene that might play an effective role in causing periodontitis. IL36RN may involve by other inflammatory cytokines in the pathogenesis of periodontitis.

## Graphical abstract

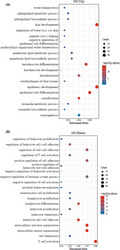

## 1 INTRODUCTION

Periodontitis, common but largely preventable, is a chronic and multifactorial inflammatory disease that damages the supporting soft tissue and bone of teeth.^1^, ^2^ Patients with periodontitis disease may last for a duration of several months or years. The interaction between periodontal pathogens and host inflammatory and immune responses is involved in the pathogenesis of periodontitis.^2^ Certain diseases, such as Crohn's disease,^3^ asthma,^4^ rheumatoid arthritis,^5^ and diabetes mellitus,^6^ were reported to increase the risk of periodontitis. Complicated immune responses in the body might be playing a critical role in the progression of tissue damagement in periodontitis.^7^, ^8^

Interleukin‐36 (IL‐36), one member of the interleukin‐1 (IL‐1) superfamily, has subfamily members known as three agonists (IL‐36α, IL‐36β, and IL‐36γ) and two antagonists (interleukin‐36 receptor antagonist (IL‐36Ra and IL‐38)).^9^ IL‐36Ra is an anti‐inflammatory mediator which takes responsibility for the tight regulation of IL‐36 signaling.^9^ The IL36RN gene encodes IL‐36Ra. Various inflammatory diseases, such as inflammatory skin disorders, Crohn's disease, and rheumatoid arthritis have increasingly connected with IL‐36 related cytokines.^10^, ^11^, ^12^

In periodontitis, IL‐1, IL‐6, IL‐17A, and tumor necrosis factor‐α (TNF‐α) known as pro‐inflammatory cytokines cause body immune responses to oral bacteria specifically called *Porphyromonas gingivalis*.^13^ Kübra et al. reported that active periodontal disease may cause downregulation of inflammasome regulators and they may increase the activity of IL‐1β in periodontal disease including periodontitis.^14^ Alexandra et al. found that IL‐36γ could be a key inflammatory player in periodontitis and its associated alveolar bone resorption and could be a therapeutic target.^2^ Patrick R. et al. checked the serum, saliva, gingival cervical fluid (GCF), and gingival biopsies of patients who suffer from inflammatory periodontal disease and found the presence of elevated levels of IL‐35.^15^ In this context, several interleukins might be acting as mutated genes in periodontitis disease and may be playing an important role in occurring and progression of periodontitis.

IL‐36 has been evaluated in diverse inflammatory diseases.^16^ However, the role of IL‐36RN, a mutated gene expression of IL‐36 in periodontitis patients with peripheral blood mononuclear cells (PBMC) and plasma remains unknown. Our study aims to find IL36RN in PBMC and plasma of the periodontitis patients and its clinical significance.

## 2 MATERIALS AND METHODS

### 2.1 Patients

A total of 194 cases of periodontitis and healthy control samples with PBMC and plasma were retrospectively accumulated from GEO‐based Affiliated Hospital of Beihua University. The patients were recruited from August 2018 to January 2020. All patients signed informed consent during their hospital stay, and the study was authorized by the ethics committee of Affiliated Hospital of Beihua University and was conducted following the Declaration of Helsinki guidelines (2018080375).

### 2.2 Inclusion and exclusion criteria

#### 2.2.1 Inclusion

Periodontitis patients: Age ≥35 years old; residual teeth ≥20 (2 teeth in each quadrant); probing depth ≥6 mm; clinical attachment loss ≥5 mm; absorption of Alveolar bone≥Ⅰdegree. Healthy control: age >18 years old; No history of periodontal disease and missing teeth; no gingival swelling, spontaneous bleeding, bleeding on probing and probing depth <3 mm.

#### 2.2.2 Exclusion

Patients suffered from systemic diseases such as diabetes and immune dysfunction. Patients who took anti‐inflammatory drugs and anti‐tumor drugs three months before treatment; Patients with oral local radiotherapy; Smoking and excessive drinking; Female in pregnancy or lactation.

### 2.3 Periodontitis severity criteria

Mild periodontitis: ≥2 interproximal sites with clinical attachment loss ≥3 mm, and ≥2 interproximal sites with probing depth ≥4 mm (not on same tooth) or one site with probing depth ≥5 mm.

Moderate periodontitis: ≥2 interproximal sites with clinical attachment loss ≥4 mm (not on same tooth), or ≥2 interproximal sites with probing depth ≥5 mm (not on same tooth).

Severe periodontitis: ≥2 interproximal sites with clinical attachment loss ≥6 mm (not on same tooth) and ≥1 interproximal site with probing depth ≥5 mm.

### 2.4 RNA isolation

Total RNA was extracted by using the solutions of phenol‐chloroform afterward managing the homogenization by guanidine isothiocyanate (Trizol RNA Preparation kit). RNA concentration evaluated by NanoDrop spectrophotometer ND1000 (NanoDrop Technologies Inc.).

### 2.5 RT‐qPCR

The total RNA in the PBMC and plasma samples after transfection was extracted using Trizol reagent according to instructions. Thereafter, the total RNA was reverse‐transcribed into cDNA by the reverse transcription kit (provided by Shanghai Sangon Biological Engineering Co., LTD). The primers used are as follows: IL36RN 5′‐F: AGGCGCCAGAGGCACCATGGAC‐3′, R: 5′‐CATCCTGTGCGTTGGCTGCC‐3′, U6 F: 5′‐GAAGGTGAAGGTCGGAGTC‐3′, R: 5′‐GAAGATGGTGATGGGATTT‐3′. PCR reaction conditions were as follows: A: Pre‐denaturation at 95℃ for 10 min; B: Denaturation 95℃ 15 s; Annealing at 60℃ for 15 s, elongation at 72℃ for 20 s, a total of 40 cycles. C: 72℃ for 15 min. The reaction is terminated at 4℃. Three replicates were set for each sample, and 2^−△△Ct^ was used for relative quantitative analysis of the data.

### 2.6 Determination of cytokines

The levels of IL‐6, TNF‐α and IL‐1β were detected with corresponding Enzyme Linked Immunosorbent Assay (Elisa) kits (ThermoFisher Scientific) according to the instruction of the kit.

### 2.7 Statistical and datasets analysis

SPSS 20.0 version (SPSS Inc.) software was utilized for all the statistical analysis of the study and data were expressed as mean ± SD. Bioinformatics tools were used to analyze heatmap, volcano map, Gene Ontology (GO) and Kyoto Encylopedia of Genes and Genomes (KEGG) enrichment pathways from the GEO database. T‐test was performed for comparing the two groups. One‐way ANOVA analysis was used to compare multiple groups. Pearson correlation was used for correlation analysis. The clinical significance of IL36RN was evaluated in PBMC and plasma by the receiver operating curve (ROC) using the area under the curve (AUC). *p* < 0.05 was considered to be representing statistically significant.

## 3 RESULTS

### 3.1 Clinical characteristics

A total of 194 participants were enrolled to evaluate IL36RN expression through PBMC and plasma samples of periodontitis (n = 97) and healthy controls (n = 97). Further, the patients and healthy controls were characterized by clinical parameters such as age, gender, bleeding on probing, oral hygiene index, pocket depth, and clinical achievement level. Among both groups, the parameters of bleeding on probing, oral hygiene index, pocket depth, and clinical achievement level were statistically significant (Table 1).

**TABLE 1:** Clinical characteristics of the periodontitis patients and healthy controls

	Healthy control	Mild periodontitis	Moderate periodontitis	Severe periodontitis	*P*‐value
Age (y)	47.11 ± 5.43	45.23 ± 5.04	48.14 ± 4.81	46.37 ± 5.21	0.4863
Gender (male/female)	50/47	19/15	18/20	16/7	
Bleeding on probing (%)	71.44 ± 12.35	8.01 ± 1.34	6.52 ± 1.23	6.17 ± 1.03	< 0.001
Oral hygiene index	3.36 ± 0.71	1.49 ± 0.31	1.21 ± 0.28	0.96 ± 0.15	< 0.001
Pocket depth (mm)	4.12 ± 0.91	0.95 ± 0.17	0.81 ± 0.27	0.73 ± 0.11	< 0.001
Clinical attachment level (mm)	3.16 ± 0.45	0.77 ± 0.19	0.21 ± 0.08	0.19 ± 0.01	< 0.001

### 3.2 Discovery of IL36RN expression in periodontitis

**FIGURE 1:**
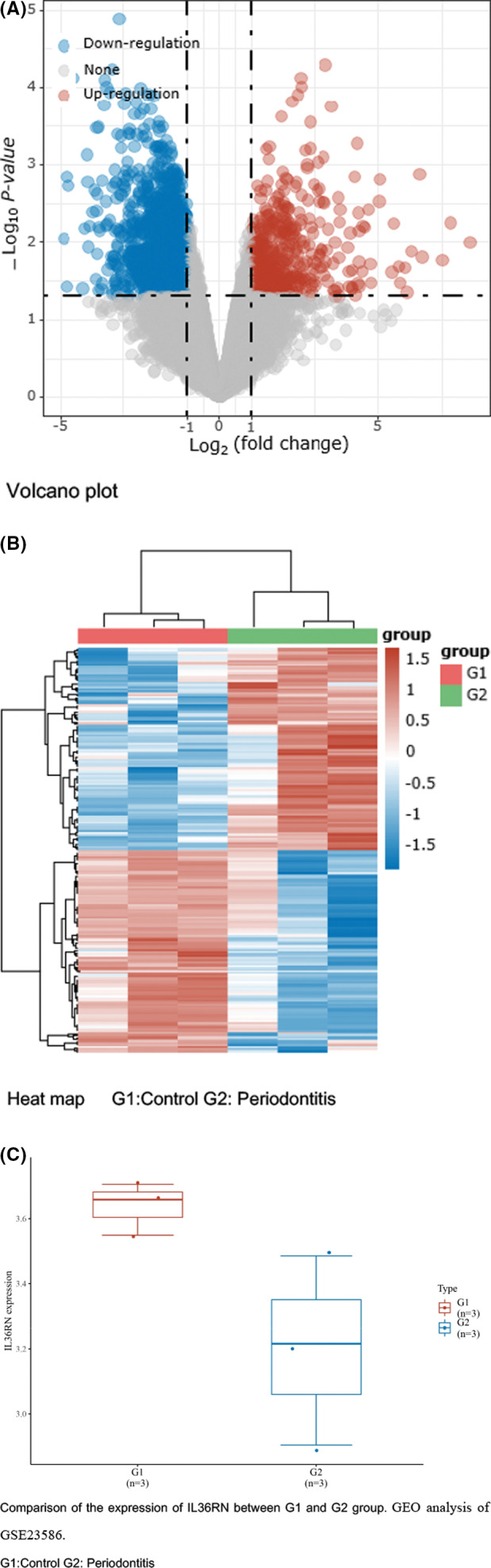
Discovery of IL36RN in periodontitis patients and healthy controls using GEO database analysis. (A) Volcano map showed significant up‐ and down‐regulated mRNA gene expression through log2‐fold change and log10 *p*‐values. (B) Heatmap clustering represented up‐ and down‐regulated expressed genes between 2 groups using fold change. (C) The comparison of IL36RN expression between 2 groups. G1: Healthy control G2: Periodontitis

Bioinformatics tools were used to discover IL36RN mutated gene in between periodontitis patients and healthy control of GEO dataset (GSE23586) via heatmap, volcano and box plot analyses (Figure 1A‐C). These heatmap and volcano plot results demonstrated that the IL36RN mutated gene was significantly down‐regulated, which was further validated by the GSE23586 dataset of periodontitis and healthy controls as per box plot (Figure 1C). In the box plot, the IL36RN expressions were lowered in periodontitis patients compared to healthy controls significantly (Figure 1C, *p* < 0.05).

**FIGURE 2:**
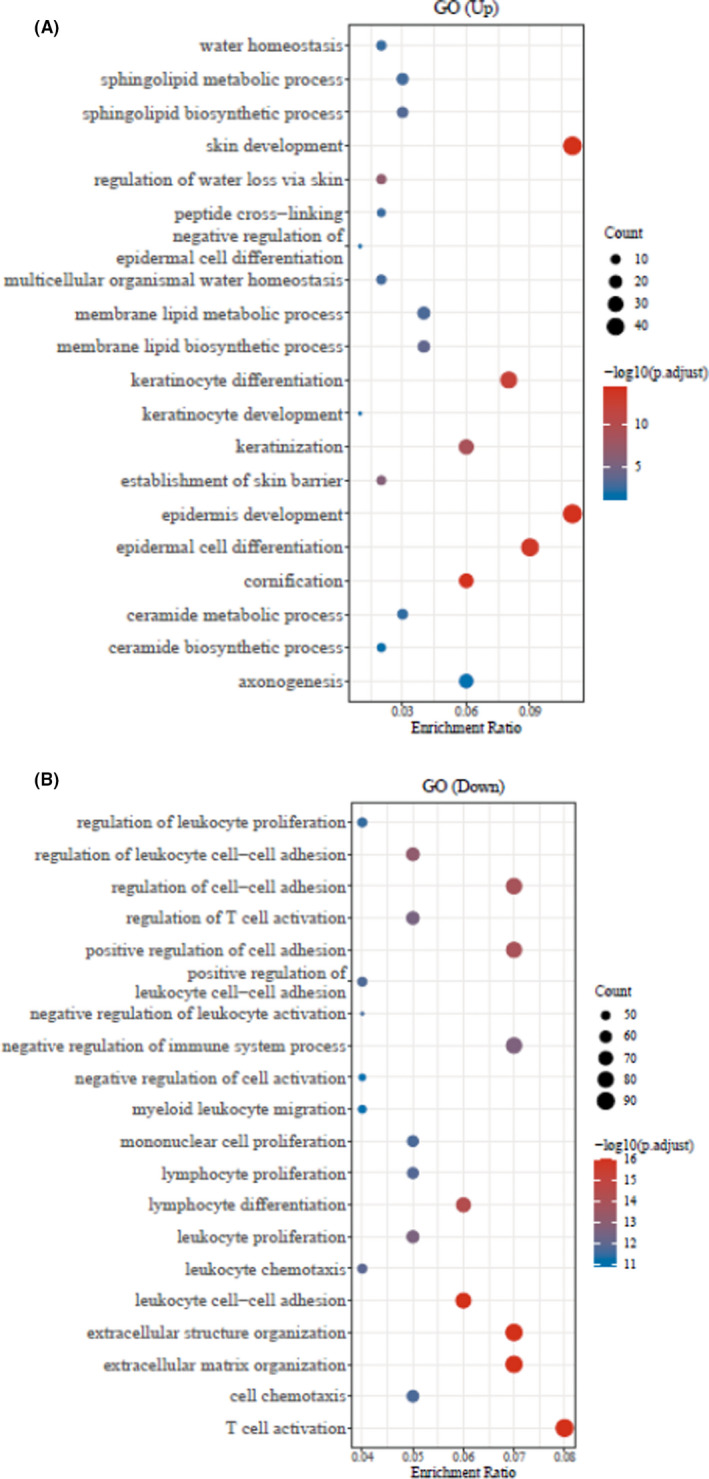
The top‐20 GO enrichment pathway analysis of up‐ and down‐regulated genes. (A) Up‐regulated GO enrichment pathways using bubble plot. (B) Down‐regulated GO enrichment pathways using bubble plot

**FIGURE 3:**
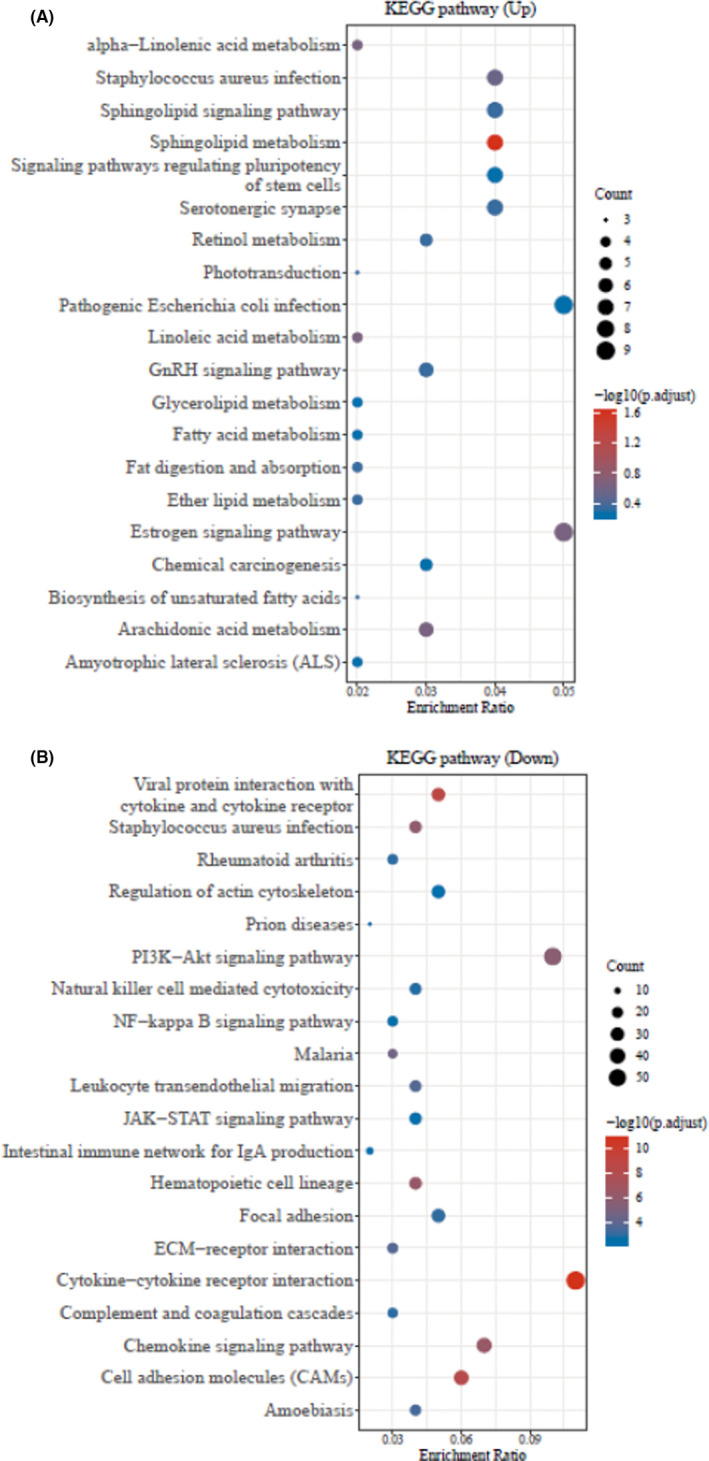
The top‐20 KEGG pathway analysis of up‐ and down‐regulated genes. (A) Up‐regulated KEGG enriched pathways using bubble plot. (B) Down‐regulated GO enrichment pathways using bubble plot

Furthermore, the GO database analysis represented the top‐20 significant up‐ and down‐regulated genes associated to GO enriched pathways of periodontitis and healthy control groups (Figure 2A,B, *p* < 0.05). Likewise, similar up‐ and down‐regulated genes related top‐20 significant KEGG pathways were observed through the KEGG pathway database (Figure 3A,B, *p* < 0.05).

### 3.3 Validation of IL36RN in PBMC and plasma of periodontitis

**FIGURE 4:**
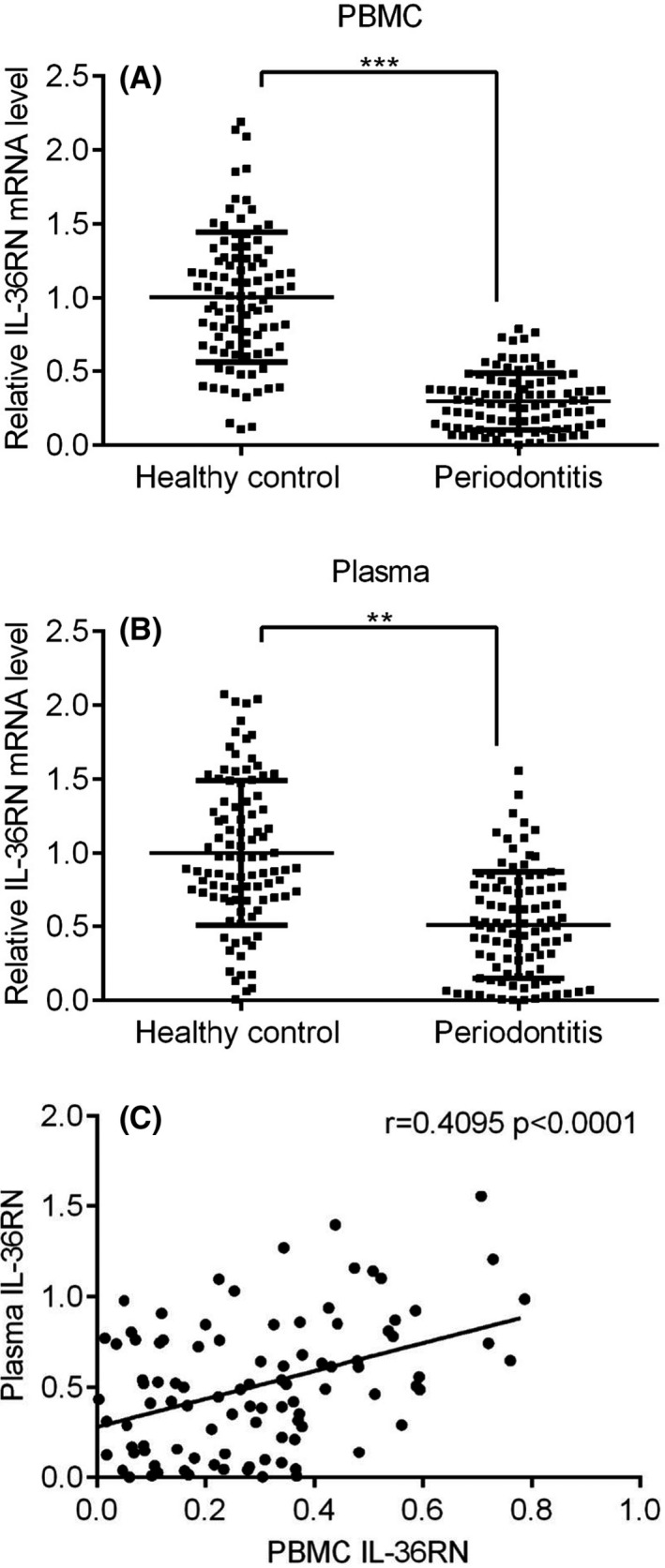
Validation of IL‐36RN mRNA expression between healthy control (n = 97) and periodontitis (n = 97) groups. (A) PBMC, (B) Plasma, (C) Correlation‐based scatter plot between PBMC and plasma

To validate between PBMC and plasma of IL36RN expressions were shown by scatter plots in 97 periodontitis patients and 97 healthy controls. Herein, IL36RN expressions were notably down‐regulated in PBMC and plasma of periodontitis patients compared to the healthy control group (Figure 4A,B, *p* < 0.05). Further, a positive correlation of IL36RN expression was significantly observed between PBMC and plasma of periodontitis patients in Figure 4C, (*r* = 0.409, *p* < 0.001) Therefore, the expression level of IL36RN in PBMC was directly correlated to plasma of periodontitis patients.

### 3.4 Three different cytokines expression in PBMC of periodontitis

**FIGURE 5:**
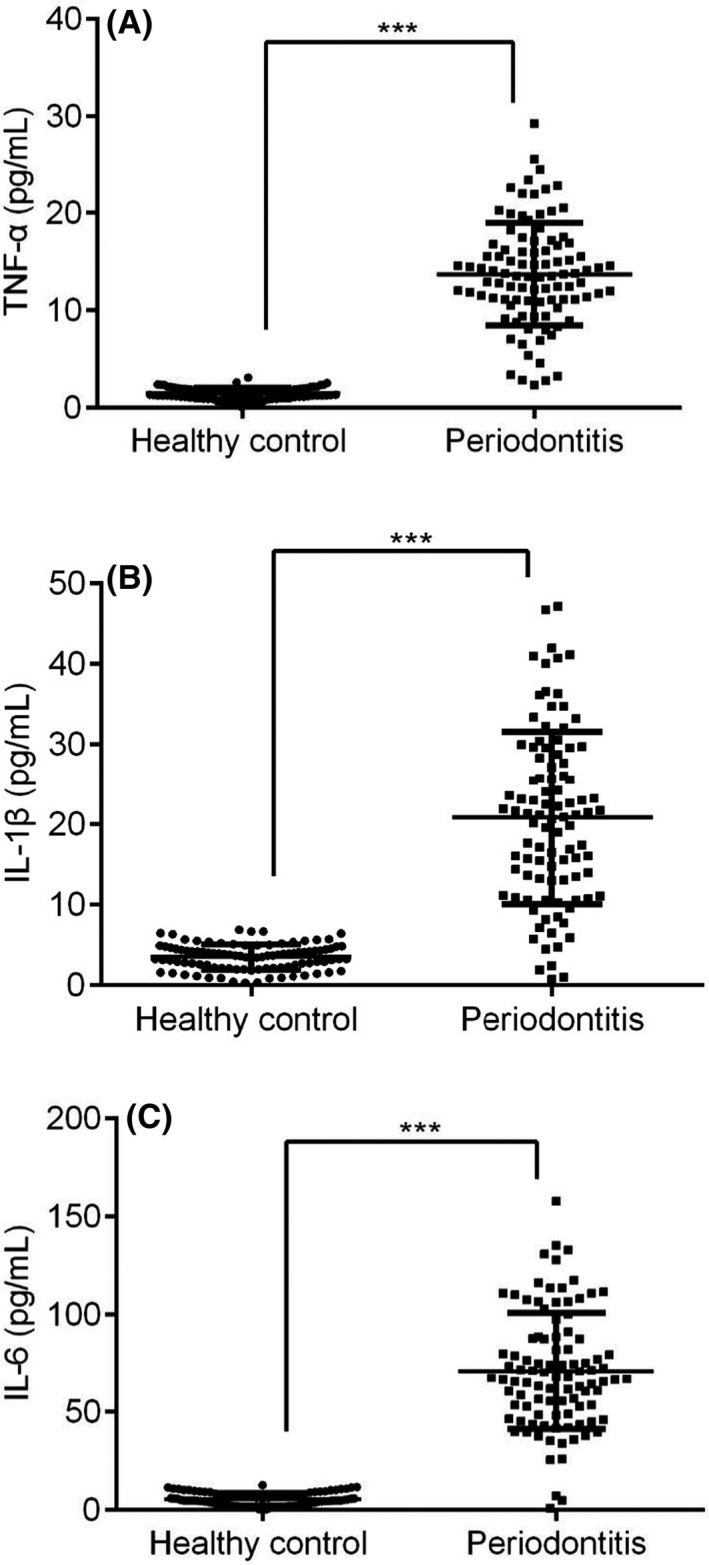
Expression of three different cytokines in serum of healthy control (n = 97) and periodontitis (n = 97) groups. (A) IL‐6, (B) TNF‐α, (C) IL‐1β

**FIGURE 6:**
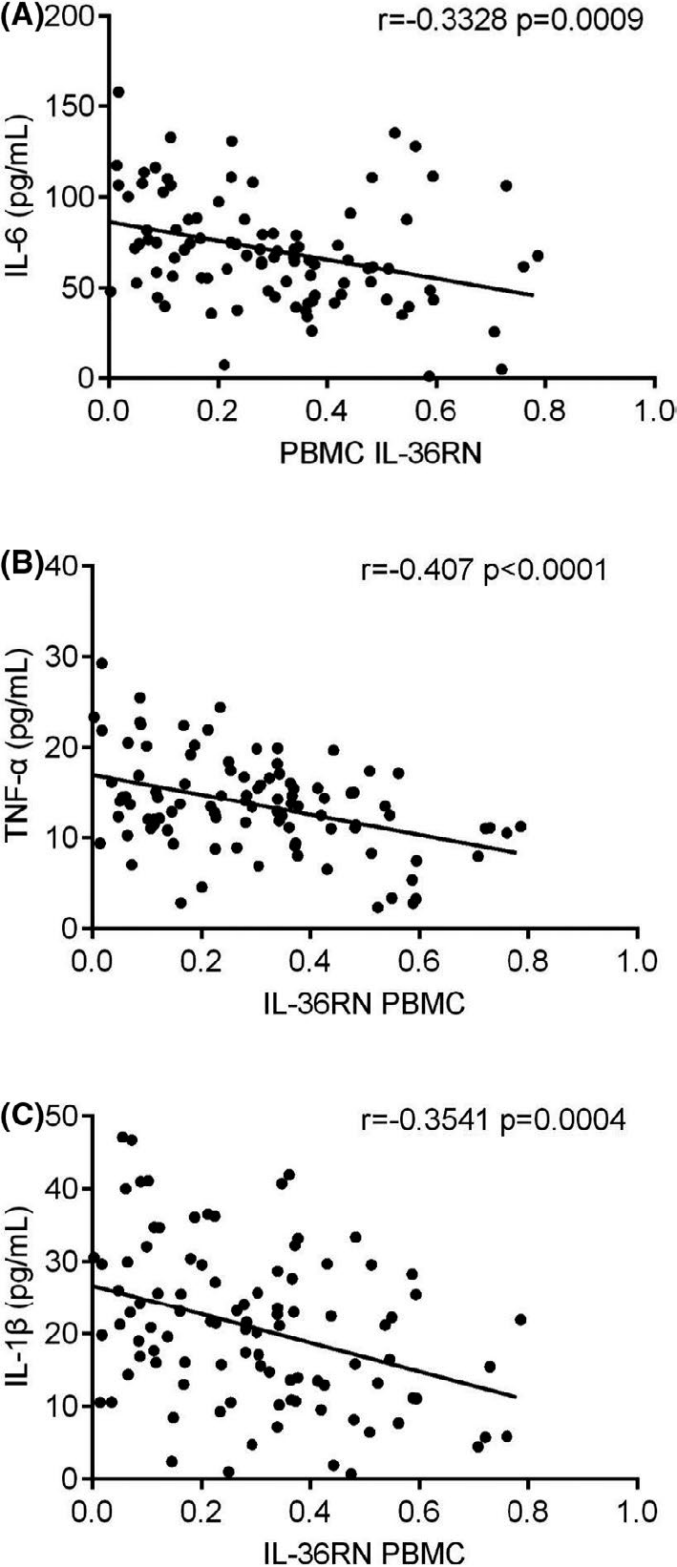
Correlation between IL‐36RN expression and serum levels of three cytokines among periodontitis patients. (A) IL‐6, (B) TNF‐α, (C) IL‐1β

Serum‐based IL‐6, TNF‐α and IL‐1β, three different cytokines expression were demonstrated in PBMC of periodontitis and healthy controls. Overall, these three different cytokines were highly expressed in periodontitis patients compared to healthy controls (Figure 5A‐C, *p* < 0.05). Meanwhile, IL36RN expression in PBMC was negatively correlated to these serum‐based three different cytokines of periodontitis patients (Figure 6A‐C, *p* < 0.05). Thus, IL‐6, TNF‐α, and IL‐1β were inversely correlated and up‐regulated expressions, which are in contrast to IL36RN.

### 3.5 Significance of IL36RN in mild, moderate, and severe periodontitis

**FIGURE 7:**
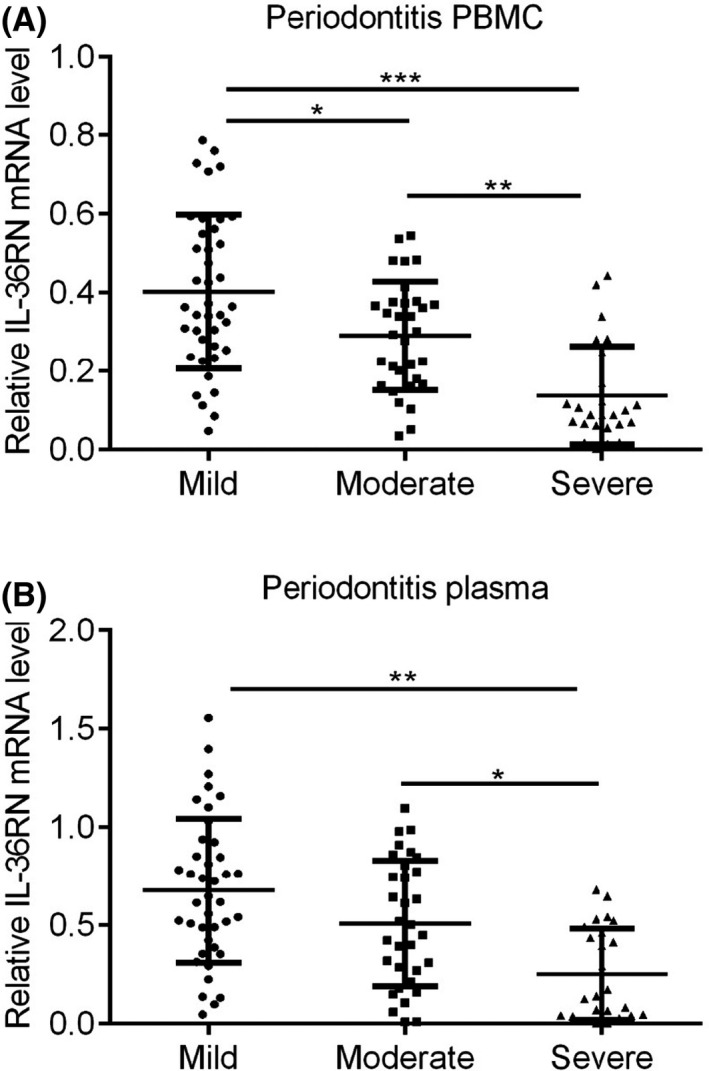
IL36RN mRNA expression in mild (n = 40) moderate (n = 32) and severe (n = 25) periodontitis patients. (A) PBMC, (B) Plasma

Besides, IL36RN expression and significance were observed in mild‐to‐severe periodontitis patients with PBMC and plasma samples. Herein, the mild periodontitis showed significantly higher expression of IL36RN in PBMC and plasma compared to moderate and severe periodontitis (Figure 7A,B, *p* < 0.05). Similarly, moderate periodontitis showed higher expression of IL36RN than severe periodontitis whereas lower expression than mild periodontitis patients (Figure 7A,B, *p* < 0.05). While severe periodontitis represented the lowest expression of IL36RN among PBMC and plasma of mild and moderate periodontitis patients (Figure 7A,B, *p* < 0.05).

**FIGURE 8:**
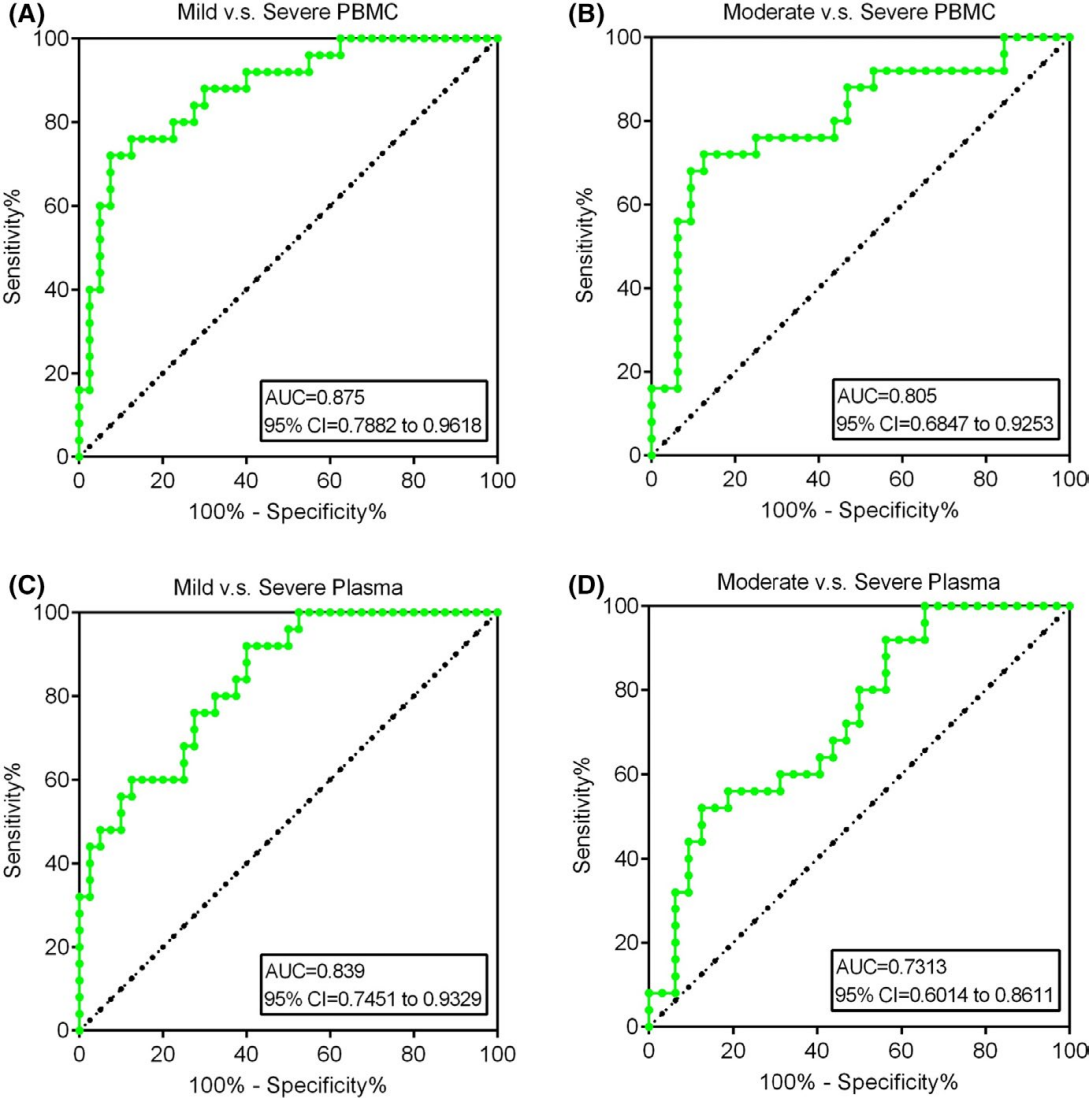
Diagnostic value of IL36RN for distinguishing mild or moderate patients from severe periodontitis patients. (A) PBMC of mild vs severe, (B) PBMC of moderate vs severe, (C) Plasma of mild vs severe, (D) Plasma of moderate vs severe

The ROC‐AUC achieved a significantly higher of 0.875 (95% confidence interval(CI) = 0.7882‐0.9618, *p* < 0.05) in mild vs severe periodontitis with PBMC whereas moderate vs severe periodontitis with PBMC achieved AUC of 0.805 (95% CI = 0.6847‐0.9253, *p* < 0.05) (Figure 8A,B). Subsequently, in plasma, mild vs severe periodontitis patients yielded AUC of 0.839 (95% CI = 0.7451‐0.9329, *p* < 0.05) while moderate to severe periodontitis patients obtained a significant AUC of 0.731 (95% CI = 0.6014‐0.8611, *p* < 0.05) (Figure 8C,D). Thus, the diagnostic value of IL36RN can accurately distinguish between mild‐to‐severe or moderate‐to‐severe periodontitis patients with PBMC and plasma, and further may provide potential significance in diagnosing periodontitis.

## 4 DISCUSSION

Periodontitis is a numerous factorial disease initially due to consisting of microorganisms based on various colonies. It is expected that occurring periodontitis is caused by environmental, genetic, and bacterial complex actions in which host factors and bacterial play a crucial role.^17^, ^18^, ^19^, ^20^ Periodontitis is linked to bleeding on probing, increased probing depth and plaque index, bone loos and clinical attachment level reduction clinically.^18^, ^19^ Bacteria of periodontal activate the host immune response that leads to the release of inflammatory cytokines and mediators in the tissues of periodontal and inducing the breakdown of periodontal.^20^ Previously, it was reported that cytokines with substantial networks play a crucial role in periodontitis pathogenesis, destroying soft tissue and resorption of bone.^21^ Besides, the existence of elevated cytokines expression comprising pro‐inflammatory and regulatory cytokines such as IL‐1β, interferon (IFN)‐γ, IL‐1 receptor antagonist (RA), IL‐4, IL‐6, IL‐10, IL‐12, TNF‐α, and induced protein (IP)‐10, lead the way to periodontitis’ inflammatory process.^22^ Thus, cytokines may involve in the pathogenesis of periodontitis.

The present study has discovered and validated the down‐regulated expression of IL36RN in periodontitis and healthy controls with PBMC and plasma samples, similar to previous studies.^19^, ^23^ Meanwhile, PBMC based IL36RN expression was positively correlated to plasma‐based IL36RN whereas the serum base three different cytokines were inversely correlated to PBMC based IL36RN. Here, the serum‐based three different cytokines (IL‐1β, IL‐6, TNF‐α) showed up‐regulated expression in periodontitis, which were consistent with previous studies.^19^, ^23^, ^24^ Moreover, previous researches mentioned that IL36RN mutation may promote general pustular psoriasis (GPP), a rare type of life‐threatening disease.^25^, ^26^, ^27^, ^28^, ^29^ Meanwhile, studies have discovered IL36RN mutations resulted in IL36‐Ra misfolded protein due to the introduction of a premature stop‐codon, frameshift mutation, or an amino acid substitution and founded that IL36‐Ra protein was poorly expressed and less stable.^25^, ^27^, ^28^ Furthermore, in the current study significance of IL36RN mutated gene was significantly distinguishing mild vs severe periodontitis and moderate vs severe periodontitis of PBMC and plasma samples with a potential AUC range of 0.73 to 0.85. Taken together, IL36RN mutation may not only affects the pathogenesis of periodontitis but also may accurately differentiate mild‐to‐severe and moderate‐to‐severe periodontitis.

In our study, closure of periodontal inflammation was obtained by removal of mechanical dental plaque, which leads to the expected betterment of periodontal infections. For few decades, most researchers have reported that non‐invasive or non‐surgical therapeutics may be a validated substitute to invasive or surgical therapeutics.^30^, ^31^ It appears that therapeutic outcomes mostly rely on the depth of probs. The periodontal pockets with 1‐3mm sizes in non‐surgical therapeutics led to 0.3mm lower losses of clinical attachment than that surgical therapeutics and reduced 0.1mm lesser depth of probes. The 4‐6mm ranging of pockets in non‐surgical therapeutics led to 0.3mm gain of more clinical attachment but reduced 0.3mm lesser depth of probes than surgical therapies. With more than 6mm pockets, surgical therapeutics represented to lead the reduction of probes depths.^32^, ^33^ Unfortunately, no efforts were made to compare the potency of different therapeutics approaches because the current study was only targeted to discover and evaluate the clinical significance of IL36RN mutated gene.

However, the study has few limitations. Firstly, the discovery of IL36RN expression was conducted by an online GEO‐based database, which might represent biased samples or findings. Secondly, the validation of IL36RN was conducted with fewer samples, thus further studies need to validate it by a larger cohort of multicenters. Thirdly, the three different cytokines were evaluated and compared using serum samples, which were not explored using PBMC or plasma‐based samples. Therefore, further studies are needed to diversely detect and evaluate IL36RN with various cytokines, which may play role in the occurrence and progression of periodontitis.

## 5 CONCLUSION

IL36RN can distinctively be detectable in periodontitis patients with PBMC and plasma, which can act as a down‐regulated mutated gene that might play an effective role in causing periodontitis. IL36RN may involve by other inflammatory cytokines in the pathogenesis of periodontitis.

## CONFLICTS OF INTERESTS

The authors declared that they have no potential conflicts of interest.

## Data Availability

The datasets used and/or analyzed during the current study are available from the corresponding author on reasonable request.
